# The “Newer Psychology”

**Published:** 1900-08

**Authors:** 


### TEXAS MEDICAL JOURNAL.

AUSTIN, TEXAS.

A MONTHLY JOURNAL OF MEDICINE AND SURGERY.

EDITED AND PUBLISHED BY

F. E. DANIEL, M. D., AND S. E. HUDSON, M. D.

Published Monthly at Austin, Texas, by Drs. Daniel and Hudson. Subscription price $1.00 a year in advance.

Eastern Representative: John Guy Monihan, St. Paul Building, 220 Broadway, New York City.

Official organ of the West Texas Medical Association, the Houston District Medical Association, the Austin District Medical Society, the Brazos Valley Medical Association, the Galveston County Medical Society, and several others.

### The "Newer Psychology,"

**HUDSON'S HERESIES.**

AN APOSTLE has arisen, preaching a new psychology. At a banquet given in New York, May 16th, by the Medico-Legal Society and its Psychological Section jointly, in honor of Thompson Jay Hudson, LL. D., an eminent lawyer of Washington City, that gentleman read a most remarkable paper, entitled "Psychological Problems Relating to Criminal Confessions of Innocent Persons." It was remarkable, in that it assumes as facts certain things that are by no means established, and claims the discovery of "laws" that are, according to the writer, in universal operation, and whereby he seeks to account for certain heretofore inexplicable mental phenomena. These "laws" are purely imaginary; or if they exist, human experience and observation in the past and present count for nothing, as they are directly in conflict therewith. They are predicated upon the assertion,—a pure assumption,—that man has two minds, one the author calls the "objective mind," and the other the "subjective mind." We will give one of these "laws" as a specimen further on. His predicate and his deductions are open to successful controversy, and it is a little surprising that such astounding statements as are contained in the paper were allowed to go unchallenged by those present. It can only be accounted for, I think, by attributing to the audience a sense of delicacy and hospitality toward their distinguished guest, for there were many there who were capable of showing the fallacy of the position and the false logic of the argument. Many able men were present,—lawyers, doctors and ministers, but there was a notable absence of medical men who are identified with mental science. The *Medico-Legal Journal* for June publishes the paper in full, with all the discussion. Nearly all who entered upon the discussion agreed with the author, and complimented him very highly. Judge Abraham Daily, of Brooklyn, a distinguished and able medico-legal scholar said that he differed with the author in many respects, but after pointing out one or two inconsistencies in the author's logic, refrained from criticism. He introduced Professor W. H. Lockwood, of Chicago, who was his guest on the occasion. The professor thought, too, that the occasion was not one for "acrimonious discussion," but he was, nevertheless, it seems, in a combative mood, and he constituted the one exception; he alone took issue with Mr. Hudson. The professor evidently has a fine sense of the ludicrous, and recognizes it even in the garb of pseudo science; he criticized Mr. Hudson's paper severely,—ridiculed some portions of it, I may say. I quote below a part of his remarks, and also some parts of the paper.

Referring to Mr. Hudson's elucidation of his postulate with regard to the "dual mind," and specifically, the "subjective mind," Professor Lockwood said:

"The author affirms that under 'the supreme potency of suggestion a subject may be made to believe himself to be a dog or a devil, a demon or an angel, the spirit of the deceased person or a living personality other than his own; and that he will carry out every suggestion to its logical conclusions so far as it is physically possible.' And yet after telling us of the carrying out of legitimate conclusions 'expressed or implied that is made to it,' he says that in this subjective state, 'Reason is dethroned, experience counts for nothing, the evidence of the senses is impeached, the centers of control over the dual mental organism is displaced; and as long as the subjective state continues or as often as it is renewed, the subject is dominated by the central idea embraced in the suggestion.' And yet, according to our friend's own words and terms, this dominated mental reaction, this mind reflex of the operator, Dr. Hudson makes one of the dual minds of the subject, for it is the 'Subjective Mind' of the party operated upon.

"Let us amplify this point. If I psychologise the Judge, and his mental fiber becomes tensioned to that point where it responds to my mental volition and 'carries out all that is expressed or implied' by the reflex of my mentality upon his—Dailey's—brain, this phenomenon, strangely enough, Dr. Hudson calls Judge Dailey's 'Subjective Mind.' In the expression of this phenomenon I will confess, that I cannot comprehend how my mental action becomes one of Dailey's dual minds, since it emanated from me. And I am still more perplexed when I recall that Brother Hudson instructs that in this expression of hypnosis, Dailey's reason is dethroned, his 'previous experience counts for nothing,' and 'the evidence of the senses is impeached,'—a most unfortunate mental plight I submit, and all the more so, since I am uncertain whether it applies to the temporary state of hypnosis which my subject is in, or to me, who made 'a vigorous effort' to control and dominate his mental fiber."

And yet, this "subjective mind," so easily "dominated," and made to believe itself "a dog or a devil," Mr. Hudson says is the *soul of man*. In a sensational book recently upblished, called "Scientific Demonstration of a Future State"—the aim of which, I gather, is to overthrow the doctrines of Huxley, and "explain the miracles recorded in the New Testament" by what he calls "the law of suggestion," (see also his book, "Laws of Psychic Phenomena), he says that of the two minds,—objective and subjective,—both functions of the brain, the latter, alone, survives the death of its organ; that it is a distinct entity, possessing independent powers and functions, and has "a mental organization" (sic) of its own, and is capable of sustaining, and does sustain an existence independently of the body. This "subjective mind" (which comprehends the instincts of procreation, self-preservation, and other faculties which man has in common with the brutes), is the *soul;* while the "objective mind,"—all the higher mental qualities,—intellectual and moral, reason, the love of truth and virtue, of justice and mercy, veneration of the Supreme Power;—those qualities that make up all that is good, great, wise and pure in man on earth, perish, he says, with the death of the brain. How, in that blissful future state, are souls to be individualized,—differentiated, having lost all distinguishing characterstics? What becomes of the "conscious Ego?"

There is no longer any doubt that anesthesia may be produced by hypnosis, and that surgical operations in that state are painless; but Mr. Hudson did not confine his "law" to the hypnotized, but makes it of universal application. We quote the following (*Medico-Legal Journal,* page 89):

"The observation and experience of every physician and surgeon will bear me out in the assertion that fear and dread, especially of imminent and inevitable death, will induce the subjective condition; and that painless death is, under this law, the rule among all sentient creatures."

And on page 90:

"It may be set down as axiomatic that Nature is ever kind to the victim of the inevitable. And this is true whether it is inevitable death or an inevitable surgical operation. Where the two conditions of imminence and inevitability are present the rule is invariable. It is Nature's compensation for her prodigality of life, and the universality of death made necessary by the process of organic evolution. The apparent cruelty of the law that all must die is mitigated in the only way possible, namely, by universal immunity from pain during the process of dissolution. And this immunity is made possible by the spontaneous induction of the subjective condition upon the near approach of the king of terrors. Even the soldier in battle experiences this immunity,—not only from pain when struck by a bullet, but from all fear of death while the battle lasts. And, if mortally wounded, he treads the inevitable path without fear and without regret.

"I think we may now safely assume that we have found the second universal law which we have been seeking, namely, the law under which a condition of mind may be induced which robs death of its terrors."

Professor Lockwood quoted a portion of the above (from page 90), and said:

"I fail to recognize this axiom or the kindness of nature to the victims of human zeal and popular ignorance. If it were true it would make these principles of hypnotism and physical nature more kind than man. If it were invariably true, it would rob Calvary and the Cross of its pathos and sentiment, it would deprive Christianity of the ideality of the story of the crucifixion of Christ, by drying up its fountain of sympathy and sentiment upon which it has built for two thousand years. It would take away from ecclesiasticism 'vicarious atonement,' and rob God himself of the 'plan of salvation;' and Christ would be made to appear only in the garb of a hypnotic, who did not suffer very much after all, for the sins of the world. No, I cannot accept this axiom; for in defiance of the mawkish mob, Bruno submitted to the torture of burning at the stake. He refused to be physchologised by priestly dogmatism, and yield up his individual manhood and opinions, although tortured by fire. Michael Servitus hurled back into the teeth of Calvin the verbal sentence which that despot tried in vain to hypnotise him into saying; fire and fagot and the horrors of mental agony did not lose to him his manhood. He defied Calvin and his system of suggestive therapeutics. The heroes of martyrdom would be lost to sight, and heroism would itself be a verbal blot upon the page of history, if nature interfered with a hypnosis for the accused victim, at the time of execution."

Mr. Hudson argues from false premises, and therefore reaches wrong conclusions. He sets up a man and then knocks him down. He asserts things to be facts that are not facts, and calls on the medical men, "every physician and surgeon present," to "bear him out."

It is not conceded by any means, that man has two minds. It is an assumption not warranted by fact, nor supported by any authority with which I am acquainted. I do not believe that well informed students of psychology will admit it; and the statement that death is always painless is contrary to the experience and observation of everybody, everywhere. It is rankly absurd, and physicians and surgeons will bear him out in no such statement as that "fear and dread, especially of imminent and inevitable death [or surgical operation], will induce the subjective condition," "a condition of mind * * * which robs death of its terror," and that in that condition death is painless; and that "under this law it is the rule with all sentient creatures." That is to say: When a man or any creature is dominated by fear of death, he does not fear death, because the fear of death puts him in that condition in which he does not fear death. Here is logic, really. And, he attributes to "Nature" this merciful provision. Let us say, then, that "whom the Gods would destroy, they first make"—anæsthetic. The absurdity of such statements is only paralleled by the story of the tender-hearted mother who, intending to spank her baby, mercifully gave it chloroform first. Were there any truth whatever in this "law," chloroform, ether and cocaine would be superfluities, and the names of Simpson, Long and Koller would have no meaning,—no place in the history of science, and none in the hearts of humanity. The stake and the rack would be a jest, and the "horrors of the inquisition" an old woman's tale.

But even were death painless, I fail to see what bearing the fact would have on that state of mind which the author says causes innocent persons to confess to crimes, and to really believe themselves guilty. He makes "analogies" of unlike things. For instance;—after propounding his newly discovered "law" that suggestion will make an innocent person (not hypnotised.—Ed.), confess to crime, and really believe that he committed it, he cites, as an illustration, the case of a man who confessed to murder, who knew and insisted that he was innocent,—but was induced to confess in the hope of pardon, which was held out to him, the circumstantial evidence being very strong against him. One is reminded here of some of Bishop Butler's "reasoning," in his once celebrated "Analogies," whereby he "proves" the existence and immortality of the soul by the "analogy" of the metamorphosis of the caterpillar into the butterfly.

Readers who are interested in psychology and especially in medical jurisprudence, will find the June number of Mr. Clark Bell's great periodical, the *Medico-Legal Journal*, intensely interesting reading, however fallacious some of the matter mentioned above may be thought to be. There can be no doubt that Mr. Hudson is very earnest and honest in the work, whatever else may be said of it.

